# Patterns of pre-operative opioid use affect the risk for complications after total joint replacement

**DOI:** 10.1038/s41598-021-01179-5

**Published:** 2021-11-11

**Authors:** Bheeshma Ravi, Daniel Pincus, Ruth Croxford, Timothy Leroux, JMichael Paterson, Gillian Hawker, Donald A. Redelmeier

**Affiliations:** 1 Division of Orthopaedic Surgery, Department of Surgery, University of Toronto, Toronto, Canada; 2 Division of Orthopaedic Surgery, Sunnybrook Health Sciences Centre, 43 Wellesley St E, Room 315, Toronto, ON M4Y 1H1 Canada; 3 Division of Orthopaedic Surgery, Toronto Western Hospital, Toronto, Canada; 4 ICES, Toronto, Canada; 5 Department of Medicine, University of Toronto, Toronto, Canada

**Keywords:** Health services, Musculoskeletal system

## Abstract

Preoperative opioid use has been shown to increase the risk for complications following total joint arthroplasty (TJA); however, these studies have not always accounted for differences in co-morbidities and socio-demographics between patients that use opioids and those that do not. They have also not accounted for the variation in degree of pre-operative use. The objective of this study was to determine if preoperative opioid use is associated with risk for surgical complications after TJA, and if this association varied by degree of use. Population-based retrospective cohort study. Older adult patients undergoing primary TJA of the hip, knee and shoulder for osteoarthritis between 2002 and 2015 in Ontario, Canada were identified. Using accepted definitions, patients were stratified into three groups according to their preoperative opioid use: no use, intermittent use and chronic use. The primary outcome was the occurrence of a composite surgical complication (surgical site infection, dislocation, revision arthroplasty) or death within a year of surgery. Intermittent and chronic users were matched separately to non-users in a 1:1 ratio, matching on TJA type plus a propensity score incorporating patient and provider factors. Overall, 108,067 patients were included in the study; 10% (N = 10,441) used opioids on a chronic basis before surgery and 35% (N = 37,668) used them intermittently. After matching, chronic pre-operative opioid use was associated with an increased risk for complications after TJA (HR 1.44, p = 0.001) relative to non-users. Overall, less than half of patients undergoing TJA used opioids in the year preceding surgery; the majority used them only intermittently. While chronic pre-operative opioid use is associated with an increased risk for complications after TJA, intermitted pre-operative use is not.

## Introduction

As the population ages, there has been an increase in the number of individuals suffering from arthritis, particularly osteoarthritis (OA)^1^. Historically, acute and chronic osteoarthritis pain has been managed using a combination of anti-inflammatories, injections, and physical therapy, and failing these, opioids^2,3^. Ultimately many patients with refractory arthritis pain will progress to total joint arthroplasty (TJA), which is generally successful at reducing pain and improving function, particularly for hip arthritis. However many patients with end-stage arthritis may be unwilling to consider arthroplasty, be unable to have one (eg: medically unwell), or may not have timely access to joint replacement (eg: wait times and insurance status). As a result, there are patients living with symptomatic OA that have failed non-opioid analgesic modalities, and have been prescribed opioids to manage their pain.

In recent years evidence has emerged to suggest that pre-operative opioid use negatively impacts outcomes and increases early complication rates following TJA^4–8^. Both OARSI and the AAOS, among some other groups, have recommended against the use of opioids for arthritis pain^9,10^. Although there are obvious benefits to decreasing opioid use among older patients, one clear criticism of the research to date is that it fails to stratify patients by how they use narcotics. Up until this point, pre-operative opioid use has been viewed in a binary way—use or non-use. This approach precludes the possibility that the pattern of opioid use before surgery is a factor in any potential increased risk for complications—therefore also precluding the possibility that a reduction in opioid use (if cessation is not possible) may mitigate these potential risks, akin to the impact of smoking reduction or cessation before surgery^11^.

Aside from the binary definition of opioid use, previous studies have also been limited by relatively small sample sizes that precluded analysis of late complications, or inadequate risk adjustment that did not fully control for the differences between opioid users and non-users. In the present study, we used a large population database to identify patients undergoing TJA of the hip, knee and shoulder, and used medication records to stratify these patients according to their pattern of opioid use based on accepted definitions: non-users, intermittent users, and chronic users. Our primary objective was to determine if the pattern of preoperative opioid use would influence post-TJA complication rates (early and late). Our hypothesis was that chronic pre-operative use of opioids would increase complication risk following TJA, but intermittent use would not.

## Methods

### Study design and data sources

We conducted a population-based cohort study utilizing administrative data from Ontario, Canada. All methods were carried out in accordance with relevant guidelines and regulations. Patients in Ontario are insured under a single-payer system, the Ontario Health Insurance Plan (OHIP) that covers all hip, knee and shoulder total joint replacements^12^. All inpatient hospital stays and same day procedures are reported in the Discharge Abstract Database (inpatient procedures) and the National Ambulatory Care Reporting System (same day surgery)^13–15^. Both databases identify the procedures performed during the hospital stay, using Canadian Classification of Health Interventions (CCI) codes, and patient comorbidities and complications, using International Statistical Classification of Diseases and Related Health Problems 10^th^ revision (ICD-10) codes. Study protocols were approved by IC/ES. Use of the data in this study was authorized under Section 45 of Ontario’s Personal Health Information Protection Act, which does not require review by a Research Ethics Board.

The Ontario Drug Benefit (ODB) funds prescription medication for all patients aged 65 years and older. The ODB database contains a record for each prescription filled including the date, physician, number of days supplied, and drug identification number (DIN).

Baseline and post-discharge covariates for each patient were obtained from the following databases: the Registered Persons Database (RPDB), for basic demographic information on each individual; the ICES Physician Database, for surgeon demographic information and specialty; the Continuing Care Reporting System, for identifying patients treated in complex continuing care facilities; and the National Rehabilitation System database, for identifying stays at inpatient rehabilitation institutions.

### Participants

**Figure 1:**
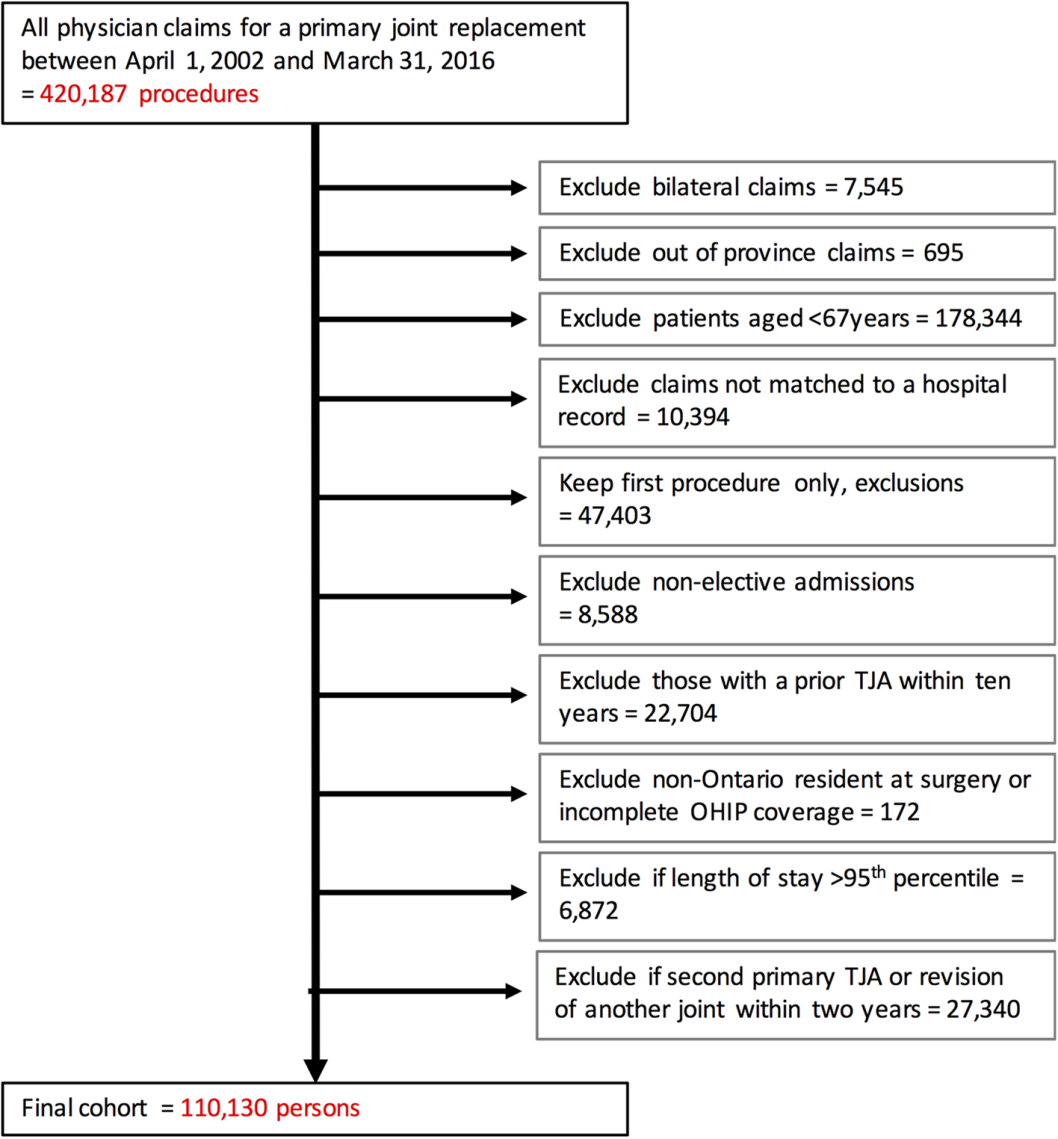
Assembling the study cohort.

We selected patients receiving elective primary total joint replacements (hip, knee and shoulder) between April 1, 2002 and March 31, 2016 from physician and hospital records. We excluded patients who were younger than 67 years (to allow for a look-back period for pre-operative opioid use), bilateral procedures, and patients from out of province. Only the first elective joint replacement for each patient was retained. Individuals were followed for 12 months after surgery. For additional exclusions, please refer to Fig. 1.

### Primary exposure

The exposure of interest was opioid use in the year immediately preceding the surgery. Opioid medications included codeine, oxycodone, hydrocodone, hydromorphone, meperidine and fentanyl. Each patient was categorized as a ‘non-user’, an ‘intermittent-user’ or a ‘chronic user’ based on their opioid use during the year before surgery. ‘Non-users’ were individuals who did not fill a prescription for opioids in the year prior to their joint replacement. ‘Chronic use’ was determined using a well-established definition, and these were individuals who had at least 90 days of continuous use of opioids in this period^16,17^. “Continuous” in this context refers to patients that received one or multiple prescriptions in succession, where the days dispensed added up to 90 days or more. Individuals who had some use but who did not meet the criteria for chronic use were categorized as ‘intermittent-users’.

### Outcome of interest

The primary outcome of interest was the occurrence of a composite complication of deep infection requiring surgery, dislocation or revision arthroplasty within one year. These complications were joint-specific—i.e. a shoulder infection did not count as a complication for someone who had a hip replacement. We used a composite outcome, as the rates of these complications at one year are generally quite low (< 0.5%)^18–20^. Additionally, we analyzed each complication individually. We additionally looked at a composite of readmission to the hospital and return to an emergency department within 30 days. These complications were identified using ICD-10 diagnostic, OHIP billing and CCI procedure codes^19^. Infections were identified by the occurrence of a hospital code for intra-articular infection with a confirmatory procedure code or physician claim for an irrigation and debridement, or a spacer insertion^18^. Revision procedures were identified using CCI codes accompanied by the supplementary status attribute “R”^18^.

### Covariates of interest

Patient age, sex and neighbourhood income quintile were obtained from the RPDB^21^. Co-morbidities in the four years before surgery were categorized using the Deyo-Charlson Index^22^ and the Elixhauser scale^23^. Frailty was defined using the Adjusted Clinical Groups (ACGs) indicator (The Johns Hopkins ACG System Version 10.0)^24,25^. Rheumatoid arthritis was identified using a previously validated algorithm^26^. Income quintile and the Ontario Marginalization Index were used as surrogates for socioeconomic status^27–30^. Obesity was determined from surgeon billing records. Surgeon volume was defined as the number of arthroplasty procedures performed by the surgeon in the previous year^19^. Hospital-volume was similarly defined. Hospitals were also categorized as either ‘academic’ or ‘community’ (www.cahohospitals.com)^31^.

### Matching and statistical analysis

Intermittent opioid users were matched to non-users by the joint being replaced (hip/knee/shoulder) and a propensity score incorporating socio-demographics (age, sex, income quintile, Ontario Marginalization Index, rurality), pre-existing health status (Charlson score, Elixhauser score, frailty, obesity, rheumatoid arthritis), provider characteristics (teaching hospital, surgeon volume, hospital volume) and the year of surgery. These patients were matched using calipers of width equal to 0.2 of the standard deviation of the logit of the propensity score^19,32^ via the greedy (or “nearest neighbor without replacement”) matching method^33^. A matching ratio of 1:1 was used^33^. Patients were specifically matched by the joint replaced, such that knee replacements in intermittent opioid-users were only being compare to knee replacements in non-users, and so on.

This process was then repeated to match chronic-users to non-users. We estimated standardized differences for all covariates before and after matching, with a standardized difference of 10% or more considered indicative of imbalance^34^. Complications were compared between the two groups using proportional hazards survival analyses adjusted for matching All analyses were performed using SAS software (version 9·3 and SAS EG 6·1, SAS Institute, Cary, NC). The two-tailed type I error probability was set to 0·05 for all analyses.

## Results

### Baseline patient characteristics

Between April 1, 2002 and March 31, 2016, we identified 110,130 eligible arthroplasty recipients (Fig. 1). Most patients (n = 60,951; 55%) did not use opioids in the year prior to their joint replacement (Table 1). Approximately 10% of the cohort (n = 10,787) were chronic users of opioids prior to surgery, and the remainder (n = 38,392; n = 35%) were intermittent-users. Relative to non-users, chronic users had a higher proportion of females (66% versus 58%, p < 0.001), and a higher number of co-morbidities.

**Table 1:** Baseline characteristics of entire cohort stratified by pre-operative opioid use.

	Non-user	Intermittent-user	Chronic user	p-value*
	N = 60,951	N = 38,392	N = 10,787	
Age (y) [Median (IQR)]	74 (70–79)	74 (70–79)	74 (70–79)	< 0.001
Female [N (%)]	35,484 (58.2%)	23,486 (61.2%)	7,148 (66.3%)	< 0.001
**Income Quintile [N (%)]**				< 0.001
Lowest	9,085 (14.9%)	6,589 (17.2%)	2,336 (21.7%)	
2	11,729 (19.3%)	7,664 (20.0%)	2,375 (22.1%)	
3	12,112 (19.9%)	7,696 (20.1%)	2,133 (19.8%)	
4	13,203 (21.7%)	7,930 (20.7%)	1,958 (18.2%)	
Highest	14,661 (24.1%)	8,411 (22.0%)	1,950 (18.1%)	
Rural [N (%)]	8,260 (13.7%)	4,723 (12.4%)	1,539 (14.4%)	< 0.001
**Joint Replaced [N (%)]**				< 0.001
Hip	21,454 (35.2%)	15,219 (39.6%)	5,023 (46.6%)	
Knee	38,504 (63.2%)	22,449 (58.5%)	5,418 (50.2%)	
Shoulder	993 (1.6%)	724 (1.9%)	346 (3.2%)	
Chronic care prior to TJA [N (%)]	184 (0.3%)	164 (0.4%)	111 (1.0%)	< 0.001
Long-term care prior to TJA [N (%)]	182 (0.3%)	215 (0.6%)	232 (2.2%)	< 0.001
**Charlson Score [N (%)]**				< 0.001
0	44,937 (73.7%)	26,510 (69.1%)	6,598 (61.2%)	
1	9,955 (16.3%)	6,952 (18.1%)	2,249 (20.8%)	
2	4,059 (6.7%)	3,100 (8.1%)	1,133 (10.5%)	
3	1,153 (1.9%)	995 (2.6%)	439 (4.1%)	
4	444 (0.7%)	428 (1.1%)	175 (1.6%)	
5 +	403 (0.7%)	407 (1.1%)	193 (1.8%)	
**Elixhauser Score [N (%)]**				< 0.001
< 0	2,162 (3.5%)	1,548 (4.0%)	557 (5.2%)	
0	48,295 (79.2%)	28,562 (74.4%)	7,296 (67.6%)	
1–4	3,718 (6.1%)	2,996 (7.8%)	1,036 (9.6%)	
5 +	6,776 (11.1%)	5,286 (13.8%)	1,898 (17.6%)	
Frailty [N (%)]	4,364 (7.2%)	3,367 (8.8%)	1,472 (13.6%)	< 0.001
Rheumatoid Arthritis [N (%)]	2,089 (3.4%)	1,778 (4.6%)	719 (6.7%)	< 0.001
Obese (billing code) [N (%)]	536 (0.9%)	392 (1.0%)	177 (1.6%)	< 0.001
Prior bariatric surgery [N (%)]	22 (0.0%)	17 (0.0%)	12 (0.1%)	0.004
Number of Aggregated Diagnosis Groups^†^ [Median (IQR)]	10 (8–13)	11 (9–14)	12 (10–15)	< 0.001
**TJA Hospital stay**				
Length of stay [Median (IQR)]	4 (3–5)	4 (3–5)	4 (3–6)	< 0.001
Long-term care after discharge [N (%)]	461 (0.8%)	386 (1.0%)	311 (2.9%)	< 0.001
**Provider demographics**				
Surgeon volume [Median (IQR)]	149 (103–208)	143 (98–198)	142 (97–200)	< 0.001
Hospital volume [Median (IQR)]	615 (408–880)	592 (390–851)	593 (388–852)	< 0.001
Teaching hospital [N (%)]	17,839 (29.3%)	10,468 (27.3%)	3,244 (30.1%)	< 0.001
**Complications within 30 days**				
Readmission or return to the ED	8,745 (14.3%)	5,812 (15.1%)	1,731 (16.0%)	< 0.001
**Complications within 1 year**				
Any surgical complication	678 (1.1%)	523 (1.4%)	218 (2.0%)	< 0.001
Infection	354 (0.6%)	249 (0.6%)	102 (0.9%)	< 0.001
Dislocation	149 (0.2%)	131 (0.3%)	74 (0.7%)	< 0.001
Revision	392 (0.6%)	293 (0.8%)	118 (1.1%)	< 0.001

*IQR* interquartile range.

*P-value testing the hypothesis of no difference among the three groups of opioid users.

^†^The Johns Hopkins ACG System Version 10.0.

### Comparing intermittent opioid-users with non-users

We successfully matched 36,250 (94%) intermittent opioid users with non-users (Table 2). After matching, absolute standardized differences were less than 10% for all measured confounders, indicating balanced (or comparable) groups. The matched analysis found no difference in the risk for our primary outcome (composite surgical complication) within one year of surgery. However, intermittent opioid users were at a slightly higher risk for readmission or return to the emergency department within 30 days (HR 1.05, p = 0.024) (Table 3).

**Table 2:** Baseline characteristics of matched cohorts*.

	Intermittent users versus non-users			Chronic opioid users versus non-users		
	Intermittent-user	Non-user	Standardized Difference	Chronic user	Non-user	Standardized Difference
	N = 36,250	N = 36,250		N = 10,279	N = 10,279	
Age (y) [Median (IQR)]	74 (70–79)	75 (71–79)	0.02	74 (70–79)	75 (71–79)	0.02
Female [N (%)]	22,064 (60.9%)	22,069 (60.9%)	0.00	6,763 (65.8%)	6,709 (65.3%)	0.01
**Income quintile [N (%)]**						
Lowest	6,047 (16.7%)	5,872 (16.2%)	0.01	2,180 (21.2%)	2,107 (20.5%)	0.02
2	7,195 (19.8%)	7,363 (20.3%)	0.01	2,273 (22.1%)	2,330 (22.7%)	0.01
3	7,283 (20.1%)	7,265 (20.0%)	0.00	2,024 (19.7%)	2,044 (19.9%)	0.00
4	7,592 (20.9%)	7,558 (20.8%)	0.00	1,896 (18.4%)	1,929 (18.8%)	0.01
Highest	8,133 (22.4%)	8,192 (22.6%)	0.00	1,906 (18.5%)	1,869 (18.2%)	0.01
Rural [N (%)]	4,481 (12.4%)	4,518 (12.5%)	0.00	1,467 (14.3%)	1,442 (14.0%)	0.01
**Joint replaced [N (%)]**						
Hip	14,150 (39.0%)	14,150 (39.0%)	0.00	4,720 (45.9%)	4,720 (45.9%)	0.00
Knee	21,445 (59.2%)	21,445 (59.2%)	0.00	5,249 (51.1%)	5,249 (51.1%)	0.00
Shoulder	655 (1.8%)	655 (1.8%)	0.00	310 (3.0%)	310 (3.0%)	0.00
Chronic care prior to TJA [N (%)]	140 (0.4%)	137 (0.4%)	0.00	83 (0.8%)	74 (0.7%)	0.01
Long-term care prior to TJA [N (%)]	151 (0.4%)	154 (0.4%)	0.00	142 (1.4%)	133 (1.3%)	0.01
**Charlson score [N (%)]**						
0	25,360 (70.0%)	25,523 (70.4%)	0.01	6,409 (62.4%)	6,379 (62.1%)	0.01
1	6,453 (17.8%)	6,351 (17.5%)	0.01	2,105 (20.5%)	2,114 (20.6%)	0.00
2	2,822 (7.8%)	2,836 (7.8%)	0.00	1,049 (10.2%)	1,091 (10.6%)	0.01
3	894 (2.5%)	844 (2.3%)	0.01	387 (3.8%)	383 (3.7%)	0.00
4	372 (1.0%)	361 (1.0%)	0.00	152 (1.5%)	152 (1.5%)	0.00
5 +	349 (1.0%)	335 (0.9%)	0.00	177 (1.7%)	160 (1.6%)	0.01
**Elixhauser score [N (%)]**						
< 0	1,421 (3.9%)	1,381 (3.8%)	0.01	513 (5.0%)	495 (4.8%)	0.01
0	27,329 (75.4%)	27,337 (75.4%)	0.00	7,084 (68.9%)	7,097 (69.0%)	0.00
1–4	2,681 (7.4%)	2,722 (7.5%)	0.00	946 (9.2%)	947 (9.2%)	0.00
5 +	4,819 (13.3%)	4,810 (13.3%)	0.00	1,736 (16.9%)	1,740 (16.9%)	0.00
Frailty [N (%)]	3,029 (8.4%)	3,009 (8.3%)	0.00	1,293 (12.6%)	1,278 (12.4%)	0.00
Rheumatoid Arthritis [N (%)]	1,576 (4.3%)	1,526 (4.2%)	0.01	649 (6.3%)	658 (6.4%)	0.00
Obese (billing code) [N (%)]	351 (1.0%)	375 (1.0%)	0.01	160 (1.6%)	151 (1.5%)	0.01
Prior bariatric surgery [N (%)]	16 (0.0%)	10 (0.0%)	0.01	11 (0.1%)	8 (0.1%)	0.01
Number of ADGs [Median (IQR)]	11 (9–14)	11 (9–14)	0.00	12 (9–14)	12 (9–14)	0.00
**TJA hospital stay**						
Length of stay [Median (IQR)]	4 (3–5)	4 (3–5)	0.00	4 (3–6)	4 (3–6)	0.06
Long-term care after discharge [N (%)]	337 (0.9%)	319 (0.9%)	0.01	230 (2.2%)	176 (1.7%)	0.04
**Provider demographics**						
Surgeon volume [Median (IQR)]	143 (99–198)	146 (101–205)	0.04	144 (97–200)	146 (99–206)	0.04
Hospital volume [Median (IQR)]	596 (394–857)	607 (405–875)	0.04	596 (390–856)	607 (394–875)	0.04
Teaching hospital [N (%)]	9,828 (27.1%)	10,622 (29.3%)	0.05	3,058 (29.7%)	3,157 (30.7%)	0.02

**Table 3:** Risk for complications after surgery.

	Intermittent users versus non-users		Chronic users versus non-users	
	HR (95% CI)	p-value	HR (95% CI)	p-value
**Any surgical complication within 1 year**	1.01 (0.89–1.15)	0.84	1.44 (1.16–1.79)	0.001
Infection	0.97 (0.81–1.16)	0.13	1.39 (1.02–1.90)	0.040
Dislocation	1.01 (0.78–1.31)	0.95	1.76 (1.18–2.63)	0.006
Revision	0.99 (0.83–1.17)	0.87	1.35 (1.01–1.80)	0.042
**Readmission or visit to ED within 30 days**	1.05 (1.01–1.09)	0.024	1.07 (1.00–1.14)	0.066

### Comparing chronic opioid users with non-users

We successfully matched 10,279 (95%) chronic opioid users with non-users (Table 2). After matching, absolute standardized differences were less than 10% for all measured confounders, indicating balanced groups. After matching, chronic opioid users were at a higher risk for our primary outcome of a composite surgical complication within one year (HR 1.44, p = 0.001). The relative increase in risk was largest for dislocation (HR 1.76, p = 0.006) (Table 3).

## Discussion

In the present study, we used one of the largest cohorts of older adults undergoing TJA of the hip, knee and shoulder to date to show that chronic pre-operative opioid use resulted in a significantly higher risk for certain complications as compared to non-users. Equally importantly, we found that intermittent opioid use was not associated with an increased risk. This study is the first to demonstrate that the pattern of pre-operative opioid use significantly influences post-operative complication risk. It is also one of the first to demonstrate that this increased risk also affects patients undergoing hip and shoulder replacements. Above all, the findings of this study suggest that the pattern of opioid use pre-operatively should be an important consideration in perioperative discussions pertaining to complication and revision risk following TJA.

Our results indicate that the short-term use of opioids (e.g.: to deal with an acute pain crisis from arthritis) will not increase the risk for future surgical complications, whereas continued use significantly increases this risk. Furthermore, it also suggests that once a patient requires the use of opioids to manage an acute crisis of arthritic pain that they would benefit from surgical consultation, if only to discuss the option of surgery. Finally, these results also suggest that a harm reduction strategy—i.e. encouraging cessation or perhaps even a reduction of opioid use to ‘intermittent’ levels should be attempted prior to surgery. However, this will require further study.

Viewing opioid use in a binary fashion (use or no use) does not appropriately capture the manner in which patients are actually taking these medications. This is an important consideration, not only for pre-operative counseling around the risks of surgery, but also for patients in acute crisis that may need short-term pain relief prior to surgery—we feel that it would be unfair to restrict the limited short-term use of opioids for these patients. We believe that our findings indicate that when a physician is considering giving a patient a prescription for opioids that it is an appropriate time to discuss the role of surgery and arrange for a surgical consultation. The authors must stress that we believe that a concerted effort to minimize or avoid opioid use for the management of pain secondary to arthritis must continue. Not only do these medications contribute to an increased risk for complications after surgery, but opioids are ineffective and dangerous for the treatment of chronic pain, such as that resulting from osteoarthritis^35^.

Over the past decade, there has been increased scrutiny of perioperative opioid use among patients undergoing TJA^10^. Studies have compared outcomes and complication risk following TJA between patients taking opioids preoperatively (‘users’) and those who did not (‘non-users’). Generally, these studies have demonstrated that pre-operative opioid use negatively impacts outcome and increases complication risk following TJA^4,5,7,8,36,37^. However, some of these studies were limited in sample size and were unable to appropriately adjust for potential confounders, including patient age and co-morbidity. The current study was one of the largest to examine the impact of opioids on complications following joint replacement in older adults, and our matching strategy (by type of joint and by a propensity score encompassing several patient and provider factors) accounted for potential confounders that may have accounted for the increased risk seen in patients that take opioids.

In our cohort only 1 out of every 5 (21.9%) patients with a history of preoperative opioid use were considered chronic users. These patients were at a 44% increased risk for any surgical complication as compared to non-users, but that the risk for individual complications was also significantly higher, with a 76% greater risk for dislocation and 35% greater risk for revision within one year. Evidence to date has suggested a link between pre-operative opioid use and early TJA revision^6,36^, but similar observations have not been made for dislocation risk. One explanation to account for a correlation between chronic preoperative opioid use and increased post-operative dislocation risk is potential fall risk, since chronic users of opioids may continue their increased opioid use for pain management following TJA that in turn may increase their susceptibility to falls^38^. Alternatively, patients who chronically use opioids pre-TJA may have more advanced radiographic OA^39^, making their primary TJA more difficult and inherently increasing the risk for dislocation and early revision; disease severity was outside of the scope of data available to us for evaluation. Interestingly, some research suggest that perioperative opioid use increases risk for infection following TJA^5^, but in the present study the risk for infection but did not reach statistical significance in a comparison that controlled for several relevant confounders.

Although our evidence would suggest that intermittent opioid use prior to TJA does not significantly increase the risk of a post-TJA complication, we did observe that intermittent users were at an increased risk for an ED visit and readmission following TJA as compared to non-users. While recognizing that this result may be a chance finding due to multiple testing, it may also point to differences in pain tolerance and management or differences in post-operative functional outcomes between intermittent and non-users, and may be an interesting topic for an independent inquiry in a new cohort. A separate report from our group has found that inadequate pain control is a common reason for return to the ED following joint replacement in this jurisdiction^40^.

This study has several limitations. First, some patient-level data was not available to us, including the severity of OA prior to TJA and surgical details such as the type of implant. Second, this study was based upon filling an opioid prescription, not patient consumption. It remains possible that some patients who filled a prescription did not consume the entire prescription, which would have most impact on our differentiation between intermittent users and non-users. Patients who filled a prescription for opioids but did not use them are still classified as ‘intermittent’ users under our definition. Our classification of ‘chronic’ users though is likely to remain very specific, as these patients typically had multiple prescriptions, which would indicate that they were taking the opioids that they were prescribed. Third, patients may have been using opioids for several reasons, not only because of pain secondary to osteoarthritis of their hips, knees or shoulders. Finally, we did not assess outcomes such as range of motion (ROM) or patient-reported outcome scores (PROMs). It remains possible that even intermittent opioid use will negatively impact these metrics.

Overall, less than half of patients undergoing TJA use opioids in the year preceding surgery, with most of these using opioids intermittently. Although chronic opioid use prior to TJA significantly increases the risk for a post-operative complication, intermittent use does not. Moving forward, it is important that physicians understand the relationship between patterns of preoperative opioid use and complication risk following TJA, and counsel prospective TJA patients accordingly. Physicians should avoid prolonged use of opioids and should consider referring patients that require opioids for urgent surgical consultation. There may also be a benefit to deferring surgery in chronic opioid users, to give them an opportunity to minimize their use before surgery.
